# The impact of a primary brain tumor diagnosis on caregivers: Insights from the patients’ perspective

**DOI:** 10.1007/s00520-024-08783-x

**Published:** 2024-08-19

**Authors:** Kelcie D. Willis, Morgan P. Reid, Amber Fox, Christopher S. Kleva, Paula Sherwood, Ashlee R. Loughan

**Affiliations:** 1 Department of Psychiatry, Center for Psychiatric Oncology, Massachusetts General Hospital, Boston, MA USA; 2 Department of Psychology, Virginia Commonwealth University, Richmond, VA USA; 3 School of Nursing, University of Pittsburgh, Pittsburgh, PA USA; 4 Division of Neuro-Oncology, Department of Neurology, VCU School of Medicine, 1201 East Marshall St, Richmond, VA 23298 USA; 5 Massey Cancer Center, VCU School of Medicine, Richmond, VA USA

**Keywords:** Primary brain tumor, Neuro-oncology, Caregiving, Supportive care, Qualitative research

## Abstract

**Purpose:**

The diagnosis of a primary brain tumor (PBT) causes significant distress for the caregiver-patient dyad, warranting increased supportive care intervention. Although researchers have previously assessed caregivers’ perceptions of their own supportive care needs, no study to date has identified how patients perceive the caregiving experience and/or patients’ recommendations for integrating supportive care of caregivers in neuro-oncology. This qualitative study examined caregiver distress as well as caregiver supportive care needs from the patients’ perspective to inform future intervention development.

**Methods:**

Adults with PBT (*N* = 15; *M*_age_ = 45; 53% female; 93% White) were divided into four, 90-min focus groups moderated by a clinical neuropsychologist. Patients responded to semi-structured interview questions regarding various supportive care needs throughout the course of disease. Each discussion was transcribed and coded using thematic content analysis and NVivo software. Inter-rater reliability was excellent (M_*Kappa*_ = 0.92, range = 0.85–0.93).

**Results:**

Seven distinct codes related to PBT caregivers emerged and were classified into two broader themes: Caregiver Impact (47% of coded content) and Caregiver Support (53% of coded content). Caregiver Impact refers to patients’ perspective of the practical and emotional demands of caregiving. Under Caregiver Support, patients cited a strong need for increased support of caregivers, including bereavement care, individual psychotherapy, and joint caregiver-patient dyad sessions.

**Conclusion:**

Patients with PBT expressed profound concerns regarding the demands of caregiving and its impact on the well-being of their loved ones. Findings emphasize the need for comprehensive dyadic support in neuro-oncology throughout the disease trajectory to enhance the overall quality-of-life for both patients and their caregivers.

## Introduction

A diagnosis of a primary brain tumor (PBT) often produces neurological, emotional, functional, and familial changes [1, 2]. Due to the location of the tumor and its targeted aggressive treatments, patients with PBT experience challenges related to seizures, fatigue, cognitive impairment, maintaining independence, and emotional distress—among other symptoms [3–6]. Moreover, a PBT can significantly shorten one’s lifespan given the current lack of curative treatments and high probability of recurrence [7, 8]. Inevitably, the combined negative sequelae of a PBT often translates to higher burden placed on the primary caregivers [9].

Extant research highlights caregivers’ perception of immense stress related to caring for a patient with PBT. Family and friend caregivers have previously reported challenges associated with learning how to quickly navigate the healthcare system, monitoring symptoms and administering medication, communicating with healthcare providers, managing new roles within the family system, and making difficult medical decisions, including advance care planning, without any preparation or training [10, 11]. Consequently, these caregivers often report feeling overwhelmed, isolated, and ill-equipped to handle these myriad responsibilities [12, 13]. Multiple studies have highlighted the pervasiveness of anxiety and depression in this population, such that caregivers often report emotional distress at higher rates than the patients they care for [14–17]. In addition, because they witness changes to their loved one’s personality and progressive declines in functioning, caregivers also endorse significant anticipatory grief and continued emotional distress during bereavement [18–20]. While this collective evidence underscores the immense distress of caregivers, it is unclear if patients with PBT are aware of how their diagnosis impacts their loved ones or if patients have any recommendations for addressing caregivers’ unmet needs.

Understanding patients’ perspectives on caregiver distress as well as their recommendations for intervention may improve future caregiver supportive care development. There is currently a paucity of evidenced-based, supportive care interventions for neuro-oncology caregivers [21–24]. Previous studies in neuro-oncology have identified potential barriers, including a lack of time/energy, need for more impromptu sessions or flexible scheduling, the belief that their emotional needs are less of priority than the patient’s needs, and a desire to receive services alongside their loved one with PBT [25–29]. Because the diagnosis of a PBT has a clear dyadic effect and may require dyadic intervention [15, 30, 31], it is important that both perspectives are considered when developing effective supportive care interventions—yet no study to date has documented the supportive care needs of caregivers from the perspective of the patient.

A recent qualitative inquiry of patients with PBT used focus groups to assess gaps in supportive care delivered to the patient across the disease trajectory [32]. From this investigation, six themes emerged, including patients’ desire for increased support of caregivers in comprehensive neuro-oncology programming. The current study explores this theme of caregiving in greater depth in order to inform future development of impactful, supportive care interventions for neuro-oncology caregivers that align with patients’ interests.

## Methods

The current study was approved by the ethics committee of Virginia Commonwealth University and Massey Cancer Center (HM20020548). Patients were recruited from a National Cancer Institute (NCI)–designated Cancer Center in January of 2021. The current study sent recruitment letters via mail or email to invite all patients who were seen in clinic over the previous year (2020) to participate. Patients were consented and enrolled in the present study if they met the following inclusion criteria: (1) PBT diagnosis (any grade) as confirmed by medical record; (2) age 18 or older; (3) access to Internet for a 90-min telehealth session; (4) ≥ 2 weeks post-surgical cranial treatment; and (5) without major cognitive impairment as measured by the Telephone Interview for Cognitive Status (TICS; > 21) [33]. Participants were then divided into four focus groups of 3–5 participants based on their availability. In line with Braun and Clarke [34], this sample size was deemed sufficient for adequately addressing the research aims of the overarching study [35, 36]. They participated in a 90-min focus group using a secure telehealth platform between February and April 2021. A licensed clinical neuropsychologist (ARL) moderated and guided discussion using semi-structured interview questions, allowing the moderator to probe and further explore participant responses while also maintaining consistency across all four groups. At the conclusion of the focus group, each participant completed a short battery of self-report quantitative questionnaires measuring psychological distress via a secure online data collection system (REDCap) [37] to further characterize the sample (see Loughan et al. [32] for a list of measures).

Focus group discussions were recorded, transcribed, and coded using a team of five trained coders and NVivo software [38]. All study team members (neuropsychologist, clinical psychology doctoral trainees, medical students) had prior experience with qualitative research methods and/or reviewed the steps for thematic content analysis detailed by Braun and Clarke [39]. The study team collaboratively developed a codebook based on the transcripts. Then, using this codebook, two study team members coded each transcript to ensure reliability. The moderator (ARL) coded all four group transcripts to support coding consistency and content integrity. All coded content was reviewed for agreement across transcripts. To support reliability, coders discussed all identified coding discrepancies after the first round of coding and re-coded content when appropriate. Inter-rater reliability was excellent across transcripts from all four focus groups (*M*_*Kappa*_ = 0.92, range = 0.85–0.93). Using SPSS [40] v27, descriptive statistics were analyzed for demographic data, medical variables, and self-reported psychological questionnaires measures [37] to characterize the participants and contextualize their responses. Detailed methodology for remaining study procedures has been previously reported [32]. The current study provides an in-depth analysis of themes specific to the patient’s discussion of caregivers.

## Results

### Sample composition

The full demographic, tumor-related, and distress characteristics of the sample are reported elsewhere [32]. Twenty patients with primary brain tumors indicated interest in the study, 18 were screened (2 were lost to follow-up after three failed contact attempts), and 17 met eligibility criteria. Fifteen participants consented and participated in the focus groups. Most participants were female (53%), White (93%), married (67%), and college educated (60%). Participants were 45 years old on average (range, 18–76). Most participants had private insurance (80%), and all had access to a primary care provider (100%). There was a relatively even distribution of diagnoses: glioblastoma (33%), oligodendroglioma (33%), and astrocytoma (27%). Most participants had high-grade tumors (73%) located in the right hemisphere (67%) and frontal lobe (67%). All participants had undergone surgical intervention, and most received radiation therapy (80%) and chemotherapy treatment (87%). Average time since diagnosis was 44 months (range, 5–178; low-grade range, 18–152; high-grade range, 5–178), with 53% of patients diagnosed within the past 2 years. Four participants had a history of progression (27%). Participants demonstrated either intact cognition (47%) or mild cognitive impairment (53%) as measured by the TICS. On average, the sample endorsed mild depression, mild generalized anxiety, moderate death anxiety, and moderate fear of cancer recurrence.

### Thematic content analysis findings

Seven codes related to the theme of “Caregivers” emerged. There was excellent inter-rater reliability across all seven codes under this specific theme (Mean Cohen’s kappa = 0.94; range, 0.90–0.97). Codes were classified into two categories: *Caregiver Impact* (codes: caregiver’s practical demands and caregiver’s emotional demands) and *Caregiver Support* (codes: need for caregiver support, need for bereavement support, and therapy structure recommendations).

#### Caregiver Impact

Nearly half (47%) of all caregiver-related content described the practical and emotional impact of the diagnosis on caregivers. Participants discussed the *practical demands* (21.6% of theme) placed on their loved ones following their diagnosis, including attending appointments, helping with treatment-related decisions, monitoring the patient’s medication, and financially supporting the patient and/or household. For some, these demands involved major shifts in family dynamics, such that the caregiver was suddenly responsible for tasks or roles within the family system that the patient was no longer able to manage. One man stated that he and his wife do not follow “traditional domestic roles” and struggled to “concede” a shift in responsibilities when his wife was required to take over the household chores (39 y/o male; astrocytoma).

Along with the practical responsibilities of caregiving for a patient with PBT, participants frequently mentioned the *emotional demands* (78.4% of theme) placed on their loved ones. Participants were prompted to consider whether emotional distress was more severe for patients or for their caregivers, and patients acknowledged the unique stress inherent to both roles as well as the stress shared by the dyad. For example, one participant stated, “I think it’s a combination of both. I have my own stress, and I know about [my stressors], and I’m prepared for them. And [my wife] has her stress, and she is prepared for hers. And neither one of us can imagine ourselves in the other’s place. And neither one of us wants to switch roles” (43 y/o male; glioblastoma). On the other hand, many patients felt that their caregiver was unequivocally more distressed: “It was a lot more distressing for my family members than for me. But I also had a lot of mental changes and brain fog…that were impacting my ability to process things for a while” (18 y/o female; astrocytoma).

Relatedly, participants noted a hypervigilance in their caregivers that reflected their elevated anxiety, which was demonstrated by caregivers constant checking for any medical or neurological changes that would suggest disease progression, a phenomenon conceptualized as “fear of cancer recurrence.” Others spoke about the social pressure placed on caregivers, who often become the primary point of contact and advocate for the patient at medical appointments or communication with other family members and friends. Beyond the primary caregiver, several participants described the emotional reactions of their children, with one participant stating, “It made my children have anxiety issues. And to this day, they are still dealing with those anxiety issues…I think because it was just so tense [at initial diagnosis]” (52 y/o female astrocytoma). See Table 1 for additional quotes for each code related to *Caregiver Impact*.

**Table 1:** Quotes related to Caregiver Impact theme

Quotes related to *Caregiver Impact* (47%)	
Practical demands (21.6% of theme)	“My wife has really, you talk about the binder, and helping you with reminders, and setting up appointments…She’s taken the bull by the horns and taken a lot of that off of me” (39 y/o male; oligodendroglioma)
	“Life is so changing for [my husband] because…I had done everything in the family. He didn’t even know how to access the bills. And all of a sudden, I couldn’t even use a computer. He didn’t even have passcodes, so all of a sudden, we shifted gears and he had to take over all of that” (52 y/o female; astrocytoma)
Emotional demands (78.4% of theme)	“They worry about every little thing…If I drink too many coffees and I have a hand tremor, they’re like ‘Are your hands shaking? Are you okay?’” (18 y/o female; astrocytoma)
	“I think it was really stressful for her kind of being the intermediary, being introverted and being suddenly someone who is communicating with everyone about my condition and whatnot” (39 y/o male; astrocytoma)
	“Physically, it’s harder for the cancer patient, but mentally and emotionally probably equal. Because they’re losing somebody, and they have no control over it either” (48 y/o female; astrocytoma)

#### Caregiver Support

The remaining coded content under the theme of caregiving regarded support (53% of coded content). A recurring idea in patients’ conversation about caregivers centered on the *need for increased caregiver support* within neuro-oncology (26.3% of theme). Participants noted that caregivers often have high levels of emotional distress from the point of diagnosis and continuing throughout the disease trajectory. As patients are grappling with their own physical and emotional turmoil, they are often unable to provide their caregivers with adequate empathy and support. Moreover, the focus among healthcare providers is often on patients’ needs, leaving the caregivers’ psychological needs neglected. In discussing the impact of her diagnosis on her husband, one participant stated, “There was nothing for him. There was no kind of, you know, support system…There’s no one there for him to be talking to” (52 y/o female; astrocytoma). Another participant stated, “[Caregivers] have to digest and process pretty intense emotions too…So they should probably get a support group too” (48 y/o female; astrocytoma). Participants suggested that counseling and support groups should be offered to caregivers upon first receiving the diagnosis, as well as throughout the disease trajectory. Additionally, participants recognized that their caregivers’ emotional journeys would be far from over once the patient has passed, noting a need for *caregiver bereavement support* (7.9% of theme). One participant referred to caregivers as “the ones that get left behind,” stating that support should be offered, “right up to [the patient’s death] and even afterwards, for them to be able to go through the grief stages, because it’s not going to really be real until it happens” (48 y/o female; astrocytoma).

While the need for caregiver support was clear, participants had differing ideas about the structure of psychological services offered to caregivers (65.8% of theme), particularly with regard to whether sessions should be *independent* (i.e., the patient and caregiver receive separate therapy/counseling; 15.8% of theme), *joint* (i.e., both the patient and caregiver receive services together; 23.7% of theme), or a *combination of independent and joint* sessions (26.3% of theme). Some participants stated that caregivers deserved to receive therapy/counseling independently of the patient so that they could more freely discuss their needs without fear of embarrassing or upsetting the patient, and vice versa. Moreover, it may not always be beneficial for the patient to hear everything that the caregiver says, as one patient stated: “I need [my caregiver] to be in a space where she can vent and I’m not there. I don’t want to hear” (39 y/o male; astrocytoma). Others saw the benefit of joint sessions involving both the patient and caregiver, stating that the therapist could assist the pair with communication skills and encourage difficult discussions between the two. Most participants advocated for a combination of the two modalities, suggesting a combination of both individual and joint sessions so that both patients’ and caregivers’ needs are addressed. One participant envisioned, “The patient has three weeks alone with a therapist, and the family member…has three weeks alone, and then at the fourth week, they come together” (48 y/o female, astrocytoma). She stated that it would be helpful for her caregiver to have a space to vent, while also having a space to share concerns with each other. See Table 2 for additional quotes for each code related to *Caregiver Support*.

**Table 2:** Quotes related to Caregiver Support theme

Quotes related to *Caregiver Support* (53%)		
Need for caregiver support (26.3% of theme)		“When you hear the diagnosis, [the caregiver] is hearing it, too. And they’re going through their own thing… When the doctor told me, I was just stunned, and I didn’t even realize that my daughter was over in the corner crying because I just sat there stunned. She was already processing, you know?” (48 y/o female; astrocytoma)
		“I’m all [my sister] has…From her immediate family, I’m it. She could have seriously used some help when [the diagnosis] happened, so I think [counseling] would be great for a family to be able to do” (43 y/o female; oligodendroglioma)
Need for bereavement support (7.9% of theme)		“I think at the beginning, when [the diagnosis] is announced, those emotions need to be handled. And then at the end, once the person passes, they need some support at that time, too” (54 y/o female; oligodendroglioma)
Therapy structure recommendations (65.8% of theme)	Independent session (15.8% of theme)	“I feel protective about it. I can be straight out honest with my therapist, and I really like being able to do that…” (76 y/o male; glioblastoma)
		“[With joint sessions, caregivers] might feel pressure [not to say certain things] because [the patient] is right there in the room” (39 y/o male; astrocytoma)
	Joint session (23.7% of theme)	“They go in the meeting together, start off with the patient and then the caregivers feed in, and they can get stuff off [their minds], and they can feed off each other” (22 y/o male; glioma)
		“Me and my daughter are so worried about what the other one’s going through…that we don’t talk to each other because we don’t want to burden each other. So, to [be in therapy with each other] is actually freeing” (48 y/o female; astrocytoma)
	Both independent and joint sessions (26.3% of theme)	“The first time that our therapist met with us, it was me and then it was [my wife] separately. And then after that, we reunited forces. That’s been good” (42 y/o male; glioblastoma)
		“[The caregivers] can come to the group with the patient, and they can hear how other people are relating to the patient…Then give [the caregivers] time to relate with other caregivers, and the patients can hear what the caregivers are talking about. So, the patients can know what the caregivers’ needs are” (22 y/o male; glioma)

## Discussion

The current qualitative study describes the patient’s perspective of the neuro-oncology caregiving experience, including patients’ input on caregiver supportive care needs, to inform future neuro-oncology comprehensive programming. This study extends our previous qualitative work [32] and allowed for a more in-depth exploration of the identified theme of caregiving. The results highlight the recognized burden as well as the lack of support of caregivers, both at initial diagnosis, throughout the disease trajectory, and following the patient’s passing. Specifically, patients with PBT demonstrated concern for the practical and emotional demands of their caregivers and strongly advocate that caregivers are provided psychosocial support for both their individual and dyadic needs across the disease trajectory. These findings mirror Sherwood et al.’s conceptual model of neuro-oncology caregivers [41], where the physical and mental impact of caregiving is attenuated with adequate resources—including supportive care interventions. We contextualize these findings and provide future recommendations for neuro-oncology programs below.

First, patients recognized both the practical and emotional challenges of caring for someone with PBT. This aligns with previous research that documents the experience of caregivers in neuro-oncology, both qualitatively and quantitatively. Caregivers similarly report difficulties managing their new roles and responsibilities, including attending to the patient’s neurologic decline, navigating the medical system, and facing changing family dynamics [42]. Unsurprisingly, these caregivers report high levels of burden and low levels of preparedness [43, 44]. In addition, numerous studies document the immense emotional distress experienced by caregivers, including increased anxiety, depression, death anxiety, and fear of cancer recurrence, across the disease trajectory [14–17, 19]. These emotional challenges naturally extend to the bereavement period, as described in a recent systematic review of four qualitative studies [20]. While further research is needed to characterize the distress of bereaved caregivers, extent qualitative research suggests they are left feeling isolated and lonely following the patient’s passing, struggle to make sense of their caregiving experience, and must reconcile many unanswered questions and regrets following the patient’s often rapid deterioration [45]. Patients with PBT have identified the bereavement period as an area of concern in a previous investigation, where they frequently endorsed fear of being a “burden” and worries about the “impact of their death on others” (p. 678) [5]. Because the burden of caregiving is a priority of patients with PBT and findings aligns with previous research with caregivers, it is clear that additional intervention is warranted.

As a result of the aforementioned burden of caregiving, patients with PBT strongly advocated for increased support of their loved ones. Not only did patients recommend interventions addressing both the practical and emotional aspects of caregiving, but they also stipulated that supportive care services should span the entire treatment trajectory, including after the patient’s passing. Additionally, participants of the current study specified that while some interventions should include both the patient and caregiver dyad, additional services that focus solely on the needs of the caregiver are necessary. Not only would individual psychotherapy allow caregivers a safe space to freely process their emotions without fear of upsetting the patient, but it would also afford patients the same luxury to process their concerns individually. A recent three-arm comparison (dyadic vs. individual caregiver vs. control) of a yoga program in PBT demonstrated the value of individual sessions; while attendance was best in the dyadic group, caregivers perceived more benefit when they completed the intervention alone [46]. Nevertheless, participants in the current study described the value of dyadic experiences after having separate spaces to explore their concerns in depth, and previous literature similarly suggests that patients with advanced cancer find dyadic interventions to be acceptable [47]. Overall, patients in the current study most frequently recommended a combination of both individual and joint sessions as this would allow the dyad to navigate difficult conversations, plan for the future together, and share their unique experiences of coping with the diagnosis [48, 49]. Further development of dyadic interventions in neuro-oncology is warranted, and future research should also understand how patients both perceive and benefit from caregivers’ participation in supportive care.

One example of a clinical practice that closely resembles the recommendations provided by patients in the current study is UCSF’s Neuro-Oncology Gordon Murray Caregiver Program [50], which is a philanthropically funded program designed to address both the practical and emotional needs of neuro-oncology caregivers throughout the disease trajectory, with special emphasis at diagnosis, transition periods, and following bereavement. Caregivers who participate in this program may receive support in a variety of ways: individually, alongside the patient, in a group, or from a peer mentor, further reflecting the results of the current study. While such a comprehensive program may not be feasible at every institution, components of the UCSF Neuro-Oncology Caregiver Program may be integrated into the standard of care across other academic medical and community centers. Moreover, the efficacy of these interventions should be evaluated for further refinement.

Looking to the larger literature, numerous systematic reviews document the paucity of evidenced-based interventions designed specifically for caregivers of patients with PBT [21–24]. Many of included studies were limited given the extent of missing data and/or attrition, protocol deviations, and lack of randomization [23]. Nevertheless, there are interventions that show promise for addressing the practical and emotional needs of caregivers. For example, CARE-IS [51]—a nurse-led educational intervention—and SmartCare [52]—a nurse-led online needs-based support program—target caregiver preparedness and feelings of mastery, respectively, in recent randomized controlled trials (RCTs). Psychological interventions for anxiety, depression, and existential distress are either still in development [53] or have yet to publish the results of their RCTs [54, 55]. There are currently no interventions for bereaved neuro-oncology caregivers to the authors knowledge [56]. In addition, the field lacks information regarding how patients perceive the impact of caregiving interventions, or how caregivers participation in supportive care impacts patient outcomes. In light of the state of this literature, there is room for continued development of population-specific, supportive care services for caregivers. In fact, the NIHR James Lind Alliance listed support of caregivers as a top research priority in neuro-oncology [57]—and participants in the current study would certainly agree.

The current study underscores the challenges experienced and supportive care needs of neuro-oncology caregivers through the lens of patients, which offers a unique perspective for future investigators as they continue to develop psychosocial interventions for both members of the dyad. The qualitative nature of this study provides rich information about how a PBT impacts those closest to the patient, making the need for improved care of caregivers especially salient. However, this study is not without limitations. First and foremost, the current study did not utilize purposeful sampling and did not stratify groups by tumor grade, age, or gender. We found that the mix of participants led for fruitful discussion, and the use of a focus group as opposed to individual interviews allowed participants to build off of one another and compare their experiences; however, individual interviews may have allowed all participants to share more in-depth information [58]. In addition, previous focus groups of patients with PBT have been successful [59, 60], and we similarly did not experience any significant issues with using a group format given: (1) we screened for major cognitive impairment, (2) groups were led by a neuro-psychologist well-versed in cognitive impairment, and (3) groups lasted 90 min, allowing for slower processing.

**Fig. 1:**
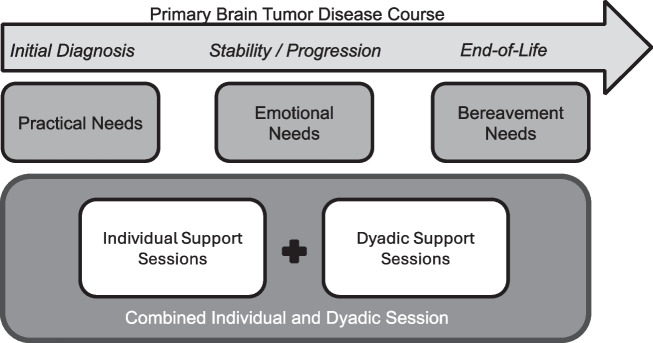
Patient recommendations for caregiver intervention

Next, while the sample included a diverse array of patients with different diagnoses, cognitive abilities, and sociodemographic characteristics, some voices were inevitably not as represented as others. Future qualitative investigations should focus on patients diagnosed with low-grade tumors specifically, as these individuals and their families may face different challenges for extended time periods compared to those with high-grade tumors [33]. Though the current study did not observe differences in tumor grade, the small representation of low-grade tumors may preclude our ability to detect differences. Future studies should also consider synthesizing the aforementioned preferences of patients with the perspective of caregivers in order to optimize future intervention development. Specifically, investigators are encouraged to characterize and quantify the distress of caregivers during bereavement, as few studies have reported on this stage of the disease trajectory [20]. Lastly, future studies should consider recruiting a community sample that does not receive care at an NCI-designated Cancer Center, as the role of the caregiver may be even more essential outside the walls of a well-resourced institution. Overall, the current study adds to the literature by demonstrating that patients understand the challenges previously documented in caregivers and believe that increased support of their loved one is an essential aspect of quality-of-life care. Moreover, this study provides valuable information about patients’ specific recommendations for treatment of their caregivers, which should be in future neuro-oncology programming (Fig. 1).

## Conclusion

This study utilized qualitative methodology to examine a theme important to patients in neuro-oncology: the care of their caregivers. Patients with PBT recognize that their loved ones face immense distress following the rapid changes tied to this life-altering and terminal diagnosis. Those who participated in the current study were concerned about both the practical and emotional demands of primary caregivers, suggesting the need for both types of intervention in neuro-oncology programs. Additionally, patients with PBT recommended that caregiver interventions are developed solely for the caregiver *and* for the patient-caregiver dyad. Lastly, patients expressed a desire for supportive care services to span across the disease trajectory, including the bereavement phase. These valuable recommendations should be considered by anyone developing patient-centric interventions in neuro-oncology moving forward. Further, given the lack of curative treatments at this time, improved supportive care that promotes the life quality of the patient should be the gold standard in neuro-oncology; and the results of the current study argue that patient-focused life quality includes the care of caregivers.

## Data Availability

Data will be made available upon reasonable request.
